# Quantitative Detection of Micro- and Nanoplastics (≥300 nm) in Human Urine Using Double-Shot Py-GC/MS with Internal Standard Calibration

**DOI:** 10.3390/toxics13060452

**Published:** 2025-05-29

**Authors:** Shanshan Ji, Wei Wang, Yong Wang, Hexiang Bai, Zhuo Li, Zongli Huo, Kai Luo

**Affiliations:** 1 Key Laboratory of Environmental Medical Engineering, Ministry of Education, School of Public Health, Southeast University, Nanjing 210009, China; jiss1118@163.com (S.J.); bhx100402@126.com (H.B.); 2 Institute of Forensic Science and Technology of Nanjing Public Security Bureau, Nanjing 210001, China; 13645198006@163.com (W.W.); wyong812@163.com (Y.W.); 3 Department of Emergency, Children’s Hospital of Nanjing Medical University, Nanjing 210008, China; lz2004@126.com; 4 Jiangsu Provincial Center for Disease Control and Prevention, Nanjing 210009, China

**Keywords:** micro- and nanoplastics, human urine, pyrolysis–gas chromatography–mass spectrometry, internal standard quantification (ISQ)

## Abstract

The rapid increase in plastic production and consumption has intensified research into human exposure to micro- and nanoplastics (MNPs) and their health effects. This study quantitatively assessed MNP internal exposure levels in non-invasive human samples, focusing on the four most common types of MNPs (≥300 nm): polyethylene terephthalate (PET), polypropylene (PP), low-density polyethylene (LDPE), and polystyrene (PS). Urine samples from 18 volunteers (4 males, 14 females) were analyzed using pyrolysis–gas chromatography–mass spectrometry (Py-GC/MS) with P(E-^13^C_2_) as an internal standard. The study developed a straightforward yet effective analytical approach for quantifying MNPs in biological fluids. MNPs were detected in all urine samples, with concentrations ranging from 0.098 to 0.986 μg/mL and an average concentration of 0.268 ± 0.235 μg/mL. LDPE, 0.074 μg/mL (interquartile range: 0.030–0.243 μg/mL), was the most abundant polymer, accounting for 67.72% of the total MNPs, followed by PS at 21.17%, while PP and PET accounted for 7.06% and 4.05%, respectively. The results also suggest that drinking water type may serve as a distinct source of MNPs in urine. This study provides novel evidence on MNP (≥300 nm) internal exposure in humans and the influence of drinking habits, highlighting the application prospects of this method in assessing the potential health risks of MNPs.

## 1. Introduction

Annually, millions of tons of plastic waste are released into the environment, driven by excessive production and consumption, alongside inadequate plastic waste management policies and practices [1,2]. Microplastics (MPs), ranging from 1 μm to 5 mm in size, and nanoplastics (NPs), which are less than 1 μm in size, are ubiquitous pollutants detected across diverse environmental matrices, encompassing marine ecosystems [3], terrestrial ecosystems [4], atmospheric systems [5], and even within organisms [6]. NPs, in particular, have garnered significant attention due to their enhanced bioavailability, extensive specific surface area, and the increased complexity associated with their removal. Toxicological studies have demonstrated that micro- and nanoplastics (MNPs) exhibit broad toxic effects on various biological groups, including plankton, fish, microorganisms, plants, and rodents [7,8,9].

While the toxicity of MNPs in humans remains poorly understood, their presence within the human body poses significant concern [10]. Numerous studies have confirmed the presence of MNPs in human samples, including blood, feces, lung, placenta, bladder, bone marrow, semen, and other body fluids or tissues [11,12,13,14,15,16,17,18]. Nonetheless, the evaluation of human exposure to MNPs remains a formidable challenge. The hydrophobic and inhomogeneous nature of MNPs results in their uneven distribution within the human body. The lack of standardized protocols for MNPs detection in human tissues or body fluid leads to difficulties in comparing results across different studies and hinders the development of a comprehensive understanding of MNP exposure and its health implications [19].

The discovery of MNPs in human blood has attracted a lot of attention as it confirmed that MNPs can enter the human circulatory system and migrate within the human body [18]. The detection of MNPs in urine holds significant importance, as it provides critical data for assessing internal exposure while offering insights into the distribution, bioavailability, bioaccumulation, and excretion mechanisms of these particles in the human body [20,21,22]. In contrast, although some studies have shown that approximately 90% of MNPs entering the human body are excreted in the feces [23], MNPs detected in the feces probably mainly represent particles that only pass through the digestive system without being significantly internalized and absorbed [20]. Moreover, as a non-invasive sampling method, urine analysis has broad applications for large-scale population exposure studies, offering reliable data to support toxicological research, public health policy formulation, and environmental risk assessment. Animal studies have revealed that both NPs (100 nm) and MPs (3 μm) are able to enter the blood and may be excreted through the urine in mice [24]. Ottavia et al. were the first to detect MPs in human kidney and urine samples [25]. Recent studies have demonstrated the presence of various types of MNPs in human urine, including polyethylene (PE), polypropylene (PP), polyethylene terephthalate (PET), polystyrene (PS), polytetrafluoroethylene (PTFE), polyvinyl chloride (PVC), polyamide 66 (PA66), polymethylmethacrylate (PMMA), polyurethane (PU), polyvinyl acetate (PVA), polyacrylamide (PAM), polycarbonate (PC), polyamide (PA), and acrylonitrile butadiene styrene (ABS), highlighting its potential as a screening target for assessing human exposure to MNPs [16,20,22,26,27].

Currently, the analysis of MNPs in human body fluids, tissues, and excreta is conducted through various methods, including spectroscopic techniques such as microanalysis-based Fourier transform infrared spectroscopy (μFTIR) and Raman spectroscopy (μRaman), alongside mass spectrometric techniques like thermal analysis-based pyrolysis–gas chromatography–mass spectrometry (Py-GC/MS) and thermo-desorption–gas chromatography–mass spectrometry (TD-GC/MS) [20,28,29]. Spectral analysis is a non-destructive test that enables qualitative analysis of the particle size, morphology, and chemical composition of MPs while preserving the intact structure. However, it has a high limitation on the size of MPs (usually > 10 μm), making it difficult to detect NPs [30]. The analysis process is susceptible to interference from the urine matrix, such as urinary salts and organic matter adhering to the microplastic surface, which affects identification accuracy [31]. Although the process of mass spectrometry leads to the loss of morphological information of MNPs, it is able to break through the particle size limitation of spectroscopic techniques and detect small-sized MPs and even NPs, greatly enhancing the comparability between different research results [32]. Compared to spectroscopic analysis, mass spectrometry is more tolerant of complex matrices and is suitable for samples rich in organic matter, such as urine [33].

However, the accuracy of quantitative mass spectrometry determinations may still be affected by matrix effects or instrumental fluctuation, and internal standard corrections need to be introduced. To address the limitations of the existing analytical methods, we focused on improving the accuracy of MNP quantification in complex biological matrices such as urine. Matrix effects in urine can significantly influence mass spectrometric signals, leading to potential errors in quantification. While isotope-labeled internal standards are commonly used to correct for such matrix interferences in other analytical fields, they have not yet been applied in the mass spectrometric analysis of microplastics in human urine. In some environmental applications, deuterated polystyrene (PS-d_5_) has been used as an internal standard [34,35]; however, it suffers from hydrogen–deuterium exchange during pyrolysis, which can affect the reliability of the identification and quantification [36]. In contrast, ^13^C-labeled polymers are more stable and provide reliable marker ions under pyrolysis conditions. Based on this, we developed a Py-GC/MS method incorporating ^13^C-labeled polyethylene [P(E-^13^C_2_)] as an internal standard to enhance the reliability and accuracy of MNP detection in urine. This methodological innovation provides a more robust and accurate tool for biomonitoring MNPsin human biological samples. This method was successfully applied to detect four types of MNPs (≥300 nm) in human urine samples. The selection of these four target substances was based on their detection frequency and concentration levels in human blood [18]. The results of this study offer valuable insights into the application of Py-GC/MS for MNP analysis and are expected to contribute to the method standardization of the human biomonitoring of MNPs, thereby enhancing data comparability across different studies and advancing our understanding of the health implications of NPs.

## 2. Materials and Methods

### 2.1. Chemical and Materials

Low-density polyethylene (LDPE, powdery, 1 to10 μm) was purchased from Alfa Aesar (Shanghai, China); polystyrene (PS, powdery, 4.8 to 5.8 μm) was purchased from Cospheric (Santa Barbara, CA, USA); polypropylene (PP, granular, 3 to 5 mm) and P(E-^13^C_2_) were purchased from Sigma Aldrich (Schnelldorf, Germany); polyethylene terephthalate (PET, granular, 3 to 5 mm) was purchased from Goodfellow (Cambridge Ltd., Huntingdon, UK); 30% hydrogen peroxide was purchased from Aladdin Reagent Co. (Shanghai, China); ultrapure water was purchased from Thermo Scientific (Optima™ LC/MS Grade, Fisher Chemical™, Waltham, MA, USA).

### 2.2. Preparation of MNP Calibration Curves

Constrained by the minimum mass limit and analytical balance error, and with the upper calibration limit being limited by the instrument, standard solutions of four polymers with internal standards were prepared by Accelerated Solvent Extraction (ASE, Thermo Scientific™ ASE™ 350, Waltham, MA, USA) according to Leslie et al. [18]. An aliquot of 2.9 to 38.4 mg of the polymer standards were weighed with a microbalance (Mettler Toledo, Columbus, OH, USA) and directly loaded into a 22 mL ASE cell. Pre-calcined diatomite (at 600 °C) was used as the dispersant and dichloromethane (DCM) as the extraction solvent. The extraction process comprised three cycles (180 °C, 1500 psi), with the combined extracts collected in a collection bottle. The extract obtained from the fourth cycle was also collected in a new bottle to estimate the extraction efficiency. The detailed parameter settings for ASE are listed in Table S1. The extracts of PP, PET, PS, and LDPE were used to prepare a mixed standard curve. The extract of P(E-^13^C_2_) was used as an internal standard. The extraction efficiency is calculated as follows:

$$Extraction\;efficiency=\frac{Peak\;area\;of\;extracts\;from\;cycles\;1\;to\;3}{The\;sum\;of\;the\;peak\;areas\;of\;extracts\;from\;cycles\;1\;to\;4}\times 100\%.$$

The standard solution was accurately measured using GC manual syringes (10 μL and 100 μL volume, part number 5190-1483 and 5190-1507, made of glass and stainless steel, Agilent, Santa Clara, CA, USA), and added directly into the pyrolysis cup, dried in an infrared drying oven, and subsequently analyzed using the Py-GC/MS system.

Due to the insoluble nature of MNPs, ensuring the stability of standard solutions is crucial for accurate quantification. In this study, the stability of standard solutions was rigorously monitored by assessing the slopes of calibration curves obtained from standard solutions analyzed over short-term (at least three consecutive days) and long-term (monthly intervals) periods. These stability assessments demonstrated that the concentrations of the standard solutions remained consistent throughout the analytical timeframe, confirming the reliability and robustness of the quantitative measurements [35].

### 2.3. Sample Collection

According to inclusion and exclusion criteria (Text S1), urine samples were collected from 18 healthy volunteers (20 to 28 years old, 4 males and 14 females) in August through September 2024. Prior to sample collection, the basic demographic information, dietary habits, and general health status of the participants were recorded using questionnaires (Table S2), with detailed information provided in Table S3. To minimize contamination during sample collection and ensure standardization and uniformity, eligible subjects were requested to strictly follow standard procedures designed specifically to prevent MNPs contamination. Participants were instructed to avoid wearing clothing made of synthetic fibers (such as polyester, PET, or blended fabrics) and to instead wear cotton clothing. Additionally, they were informed to refrain from using personal care products, including lotions, creams, deodorants, or other cosmetic products, on the day of sample collection. Prior to providing samples, all subjects were required to thoroughly wash and dry their hands. Also, the subjects were required to provide more than 10 mL of the midstream portion of their first morning urine during collection to ensure that the concentrations of various components were relatively stable. Simultaneously with sampling, several randomly selected participants were requested to collect process blank samples by opening pre-calcined glass containers and filling them with filtered ultrapure water in the same sampling environment, representing the maximal feasible simulation of potential contamination during the sampling procedure. The collected urine samples were stored in pre-calcined covered glass containers at 4 °C for further processing. This study was approved by the Ethics Committee of Children’s Hospital of Nanjing Medical University (No. 202407003-1, approval date: 22 August 2024). All subjects participating in this study provided their informed consent.

### 2.4. Pre-Processing of Samples

Urine samples, preserved in glass bottles, were thoroughly homogenized on a Heidolph™ Multi Reax Vortex Mixer (Heidolph, Schwabach, Germany) for a duration of 20 min. Then, 10 mL of the urine sample was immediately transferred into a pre-calcined 20 mL glass vial (6ASV20-1, Thermo Scientific, Waltham, MA, USA) using a glass pipette (pre-washed by ultrapure water immediately prior to use, PIPET NSTL Fisherbrand, Thermo Scientific, Waltham, MA, USA). To avoid destroying MNPs in the urine sample, a mild method was adapted in this study for the pre-processing of urine samples. Briefly, a 5 mL aliquot of Tris-HCl buffer (400 mM Tris-HCl, pH 8, 0.5% SDS, Trizbase T6791, HCl H1758, Sigma, Schnelldorf, Germany) was added to the urine sample, and the mixtures were heated at 60 °C for 1 h to denature the protein. Following this, 100 μL of proteinase K (1 mg/mL, 3.0–15.0 unit/mg, Sigma-Aldrich, Schnelldorf, Germany) and 1 mL of calcium chloride (5 mM, Aladdin, Shanghai, China) were added, and the vials were oscillated at 100 rpm in a water bath at 50 °C for 2 h. The sample vials were subsequently shaken at room temperature for 20 min and heated again at 60 °C for 20 min. Finally, titration with formic acid (FA, Sigma-Aldrich, Schnelldorf, Germany) was employed to eliminate precipitates, including phosphates and carbonates, from the urine before subsequent filtration.

To fully accommodate the folded filter membrane within an 80 μL pyrolysis cup, we customized a micro-quartz filtration tube with a diameter of 8 mm for small filter membranes (Figure S1). This filtration device ensured that the filtrate could be concentrated in the center of the membrane to reduce the loss of particulate matter to be measured during membrane folding. Prior to use, the glass fiber filter membrane (GF-75 0.3 μm, ADVANTEC, Tokyo, Japan) and the micro-quartz filtration tube were calcined at 500 °C and 600 °C for one hour, respectively, to remove any potential plastic contamination. In order to minimize the loss of MNPs by adhesion during filtration, 30% H_2_O_2_ and ultrapure water were used to rinse the inner wall of the filtration tube. The center part of the filter membrane with a diameter of 8 mm, which contained the MNP residue, was then cut out using a ring blade and carefully folded into the pre-calcined pyrolysis cup. The pyrolysis cup was then placed in a custom-made quartz cup holder (Figure S2) with a quartz cover and dried in an infrared drying oven (401, Juchuang, Qingdao, China) at 60 °C for 8 h. Upon complete drying, 20 μL of tetramethylammonium hydroxide (25% in MeOH, 334901, Sigma, Schnelldorf, Germany) was introduced as a derivatization reagent. This process induced PET to be converted to the main pyrolysis product dimethyl terephthalate (DMT, dimethyl terephthalate). Additionally, 20 μL of P(E-^13^C_2_) solution (0.0193 μg/μL) was added as an internal standard for calibration. The sample was dried again under the same conditions.

### 2.5. Analysis by Py-GC/MS

The Py-GC/MS system was constructed by integrating a multi-functional pyrolyzer (EGA/PY-3030D, Frontier, Koriyama, Japan) with an Agilent 7890A gas chromatograph (Agilent, Santa Clara, CA, USA) connected to an Agilent 5975C mass spectrometer (Agilent, Santa Clara, CA, USA). The GC was equipped with a 0.25 mm i.d. × 0.25 μm film thickness × 30 m DB5-MS column (Agilent Technologies, Palo Alto, CA, USA). A double-shot pyrolysis protocol was employed using the portable injector of the pyrolyzer. In the first stage, the pyrolysis temperature was set to a maximum of 300 °C, during which unpolymerized monomers, additives, and adsorbed substances were thermally desorbed from the polymers. Additionally, PET underwent partial degradation to generate pyrolysis products at this stage. The second stage involved heating to a maximum temperature of 600 °C, at which point pyrolysis products were generated for all four polymer types. The products from both stages were introduced into the GC/MS system. For quantitative analysis, results from both pyrolysis stages were combined for PET, whereas the second stage alone was used for the quantification of PP, LDPE, and PS. Detailed parameter settings for the Py-GC/MS system can be found in Table S4. Mass spectrometric detection was conducted in selected ion monitoring (SIM) mode. The characteristic pyrolysis product and their qualitative and quantitative ions for each analyzed polymer are summarized in Table S5. To minimize potential interference from sample matrices such as lipids during the quantification of LDPE, we adopted an approach analogous to the quantification of organic substances described previously [37]. Specifically, five homologous series of LDPE pyrolysis products, ranging from C7 to C26, were simultaneously monitored to confirm the presence of LDPE. During the quantification of LDPE, 1-hexadecene (C16), a characteristic pyrolysis product of LDPE, was selected because its longer carbon chain reduces matrix interference compared to shorter-chain pyrolysis products, thereby improving the accuracy of LDPE quantification [20].

### 2.6. Quality Assurance/Quality Control (QA/QC)

The experiment was conducted under a “plastic-free” protocol, ensuring the reliability of this study. Throughout the process, cotton lab coats and nitrile gloves were worn while working in a fume hood. The reagents used in this study were filtered using pre-calcined glass fiber filters with a pore size of 0.3 μm. Prior to filtration, all glassware was heated in a muffle furnace (SX2-4-12A, Kejing Material Technology Co., Ltd., Hefei, China) at 600 °C for 1 h to eliminate potential plastic contamination. Before every pyrolysis analysis, the pyrolysis cup and tweezers were directly burned for 3 to 5 s using a butane torch (Model 2200, Dremel, Mount Prospect, IL, USA) with an external flame temperature of 1200 °C. During the experiment, samples and contact experimental materials were encased in tin foil to prevent potential MNP contamination from the air. No plastic laboratory equipment, such as tweezers, containers, or pipettes, were used; instead, stainless steel or glass alternatives were employed.

#### 2.6.1. Recovery Experiment

The morphology (e.g., fibrous, particulate, sheet-like, or other irregular shapes), particle size, and heterogeneity of MNPs within samples significantly influence the recovery during sample pretreatment. However, these characteristics are often unknown in real-world samples. Consequently, unlike fully dissolved chemicals in liquid samples, spiking experiments with particles of specific morphologies and sizes do not reliably evaluate the recovery rate of MNPs in the pretreatment process. Despite this limitation, we estimated the approximate recovery of the filtration and drying process in this study by performing spiking experiments using ultrapure water. The recovery was validated for four target polymers. High-dose (60 μL mixed standard solutions, containing 0.2620 μg PET, 0.4608 μg PP, 0.3720 μg LDPE, and 0.3840 μg PS) and low-dose (30 μL mixed standard solutions, containing 0.1310 μg PET, 0.2304 μg PP, 0.1860 μg LDPE, and 0.1920 μg PS) standard solutions were accurately measured using GC manual syringes, transferred to glass bottles (n = 3), and concentrated using a SpeedVac™ vacuum concentrator (SPD210P1-230, Thermo Fisher Scientific, Waltham, MA, USA). Six equal volumes of ultrapure water were used as procedural blanks to monitor background contamination. A total of 12 samples were processed following the described pretreatment and analysis steps to evaluate the spiked recovery.

#### 2.6.2. Blank Control

In this study, a rigorous blank control procedure was implemented to assess potential contamination and interference throughout the MNP detection process, validating the authenticity and reliability of the test results. During the sampling phase, subjects were not only required to strictly adhere to the urine sampling protocol, but also randomly selected to collect blank samples within the same urine collection environment. This approach closely simulates the actual urine sampling procedure and allows for the simultaneous monitoring of potential contamination introduced by both the sampling environment and the subjects themselves. These blank samples underwent all the same preservation, transportation, and analytical procedures as the urine samples to evaluate the impact of environmental factors and procedural steps on the accuracy of the results. To monitor background contamination, procedural blanks were also analyzed along with the urine samples, ensuring that any contamination from the environment or the analytical process itself was identified and quantified.

#### 2.6.3. Limit of Detection (LOD) and Limit of Quantification (LOQ)

The limit of detection (LOD) and the limit of quantification (LOQ) for this method were determined as three times and ten times the standard deviation of the mean value of procedural blanks, respectively.

### 2.7. Statistical Analysis

Statistical analysis and data visualizations were performed using OriginPro 10.1.0, GraphPad Prism 9.5.0 and IBM SPSS statistics 27. Continuous demographic variables were presented as means with standard deviations (SDs), while categorical variables were reported as frequencies with percentages. Spearman’s rank correlation was employed to assess the relationship between each variable and MNP exposure abundance. A two-tailed *p*-value of <0.05 was considered statistically significant.

## 3. Results

### 3.1. QA/QC

The extraction efficiency of the four target polymers by ASE calculated after internal standard correction was 98.21–100%, showing that this method has high extraction performance for these four polymers. The calibration curves for PP, PET, PS, and LDPE were established through linear fitting using P(E-^13^C_2_) as the internal standard, covering a linear range of 22–3840 ng (Table S6). The short-term stability of the standard solutions was evaluated immediately after extraction over three consecutive days, while their long-term stability was assessed by recalibrating the standard solution concentrations every 15 days throughout two-month. As shown in Table S6, the relative standard deviations (RSDs) of the slopes of calibration curves ranged from 6.50% to 20.68%. This is an acceptable range for MNP analysis using the current technology, indicating the acceptable stability of the standard solutions throughout the entire analytical timeframe.

Six ultrapure water blanks were generated to monitor background contamination throughout the recovery test. MNP abundance in spiked samples was adjusted by subtracting the blank control values. As shown in Table 1, the recovery of low-dose spiked polymers ranged from 59.84 ± 3.09% (PP) to 117.64 ± 6.64% (PET), with a coefficient of variation of 5.16–15.67%. In comparison, the recovery of high-dose spiked polymers ranged from 56.73 ± 17.64% (PP) to 85.95 ± 13.43% (PET), with a coefficient of variation of 0.86–31.09%.

Despite rigorous quality control measures, trace concentrations of the target polymers were still detectable in procedural blanks (Table 2). To mitigate the influence of background contamination, the method limits of detection (LODs) and method limits of quantification (LOQs) were established as three and ten times the standard deviation of the procedural blanks, respectively, in accordance with ISO 11843-5:2008 [38]. Table 3 lists the instrument detection limits (IDLs), instrument quantification limits (IQLs), as well as LODs and LOQs for the four target polymers.

**Figure 1:**
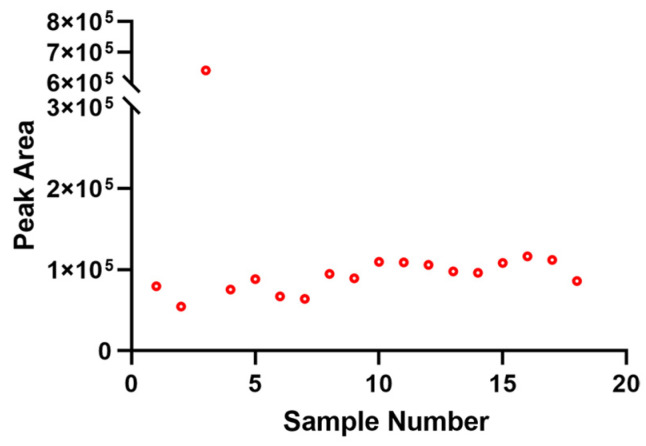
Peak area of internal standard in 18 samples.

P(E-^13^C_2_) was used as the internal standard to monitor the matrix effect and signal fluctuations in the mass spectrometer. As illustrated in Figure 1, the relative standard deviation of the internal standard peak area for the 18 samples was 20.04%, after removing one outlier by statistical methods (z-score = 3.97). This result suggests that the mass spectrometry signal fluctuates within a defined range, and applying internal standard correction can enhance the accuracy and precision of the analytical results.

### 3.2. Measured Abundance of MNPs in Urine Samples

The participants were students or teachers, all of whom worked in school environments. The architectural structures and decorative materials within the school premises were relatively uniform, thereby minimizing variability in exposure levels among individuals caused by indoor and outdoor airborne MNP pollution.

**Figure 2:**
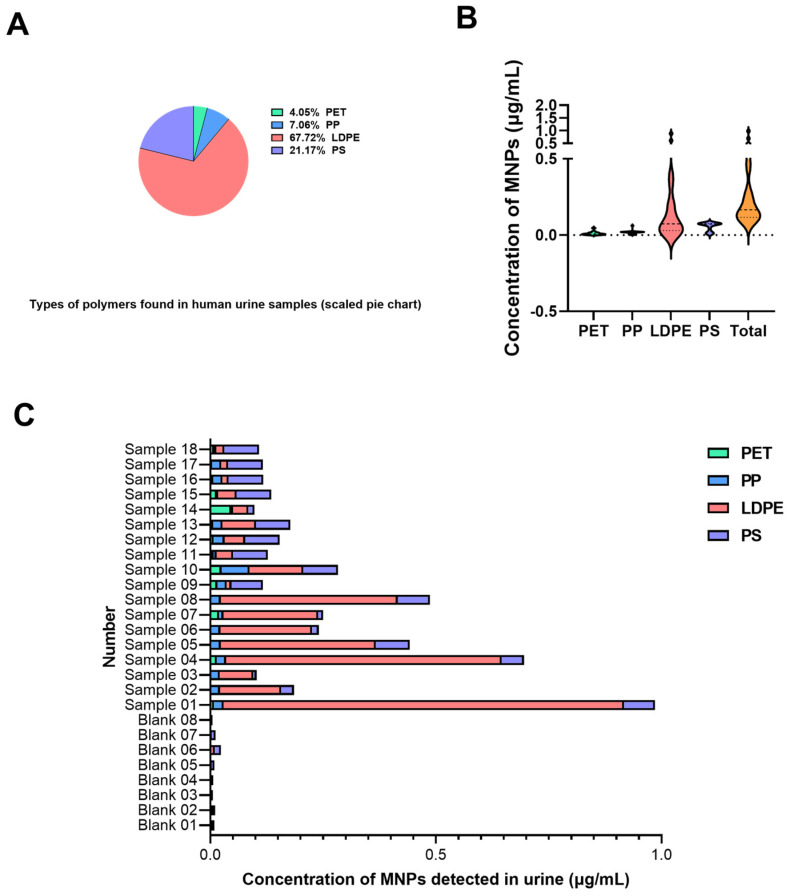
Abundance analysis of MNPs in human urine. (**A**) Types of polymers found in human urine samples (scaled pie chart). (**B**) Concentrations of MNPs in human urine samples (μg/mL) (type-based). (**C**) Comparison of total concentrations and inter-individual variations of four target MNPs detected in procedural blank controls and the urine of donors.

The urinary concentrations of MNPs among all participants (sample signals exceed blank levels significantly) are illustrated in Figure 2. All 18 samples contained at least two distinct polymer types, suggesting frequent exposure to various MNPs in daily life. The total MNP concentration ranged from 0.098 to 0.986 μg/mL, with a median of 0.166 μg/mL (interquartile range: 0.117–0.322 μg/mL) and a mean of 0.267 ± 0.235 μg/mL (mean ± SD). The data did not follow a normal distribution (the range of four polymers and total MNPs’ *w* is from 0.682 to 0.778, *p* < 0.001). When categorized by polymer type, PET and LDPE had the highest detection frequency (100%), followed by PS (55.56%), and PP had the lowest detection frequency (44.44%). In terms of measured concentrations, LDPE exhibited the highest median level at 0.074 μg/mL (interquartile range: 0.030–0.243 μg/mL), accounting for 67.72% of the total MNPs detected across all samples. This was followed by PS (median: 0.073 μg/mL, interquartile range: 0.025–0.077 μg/mL; proportion: 21.17%), PP (median: 0.021 μg/mL, interquartile range: 0.009–0.021 μg/mL; proportion: 7.06%), and PET (median: 0.008 μg/mL, interquartile range: 0.003–0.015 μg/mL; proportion: 4.05%). These findings suggest that LDPE is the predominant type of MNP excreted in participant urine samples, likely reflecting the widespread use of polyethylene-based materials in consumer products. 

### 3.3. Correlation Analysis

Since dietary habits are considered, the primary factor influencing MNP intake after excluding occupational exposure, a questionnaire survey was conducted among 18 participants to analyze factors associated with MNP abundance in urine within the general population. Specifically, we investigated participants’ intravenous injection, exercise frequency, alcohol consumption, take-out food intake frequency, bottled beverage consumption, teabag usage (including disposable paper cup-packaged milk tea drinks), aquatic product (fish) consumption, drinking water intake, drinking water type, and the material composition of tableware and cups.

**Figure 3:**
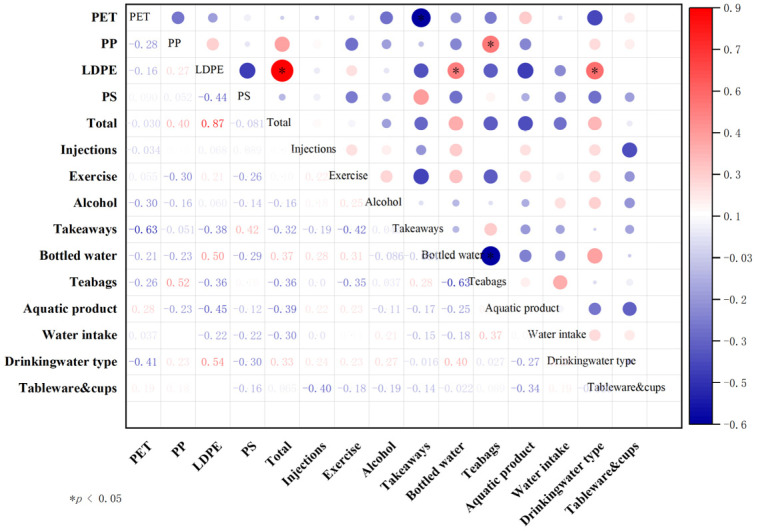
Spearman’s correlation between dietary and behavioral factor and urinary MNP abundance.

Spearman’s correlation analysis was performed to examine the relationship between each dietary and behavioral factor and MNP abundance. Figure 3 demonstrates that the detection levels of LDPE were significantly correlated with drinking water type (*p* < 0.05, r = 0.54) and the frequency of drinking bottled water (*p* < 0.05, r = 0.50). Participants who consumed bottled water daily exhibited higher LDPE detection levels than those who primarily drank filtered or boiled water. Meanwhile, the detected levels of PP exhibited a positive correlation with the frequency of teabag usage (*p* < 0.05, r = 0.52).

Furthermore, we conducted stratified analyses based on the results of the initial Spearman correlation, while accounting for gender (female/male) and body mass index (BMI, BMI < 18.5 is considered underweight, 18.5 ≤ BMI < 24.0 is normal, 24.0 ≤ BMI < 28.0 is overweight, and BMI ≥ 28.0 is obese) as potential confounders. Specifically, we examined the associations between LDPE concentrations and both bottled water consumption frequency and type of drinking water, and PP concentrations and teabag usage frequency. The results showed that, after stratification, only the type of drinking water remained significantly associated with urinary LDPE levels (*p* = 0.035), suggesting a potential link that warrants further investigation. See Table 4.

## 4. Discussion

Urine has been recognized as a reliable biological specimen for monitoring exogenous particulate matter in the human body, including virus particles, engineered metal nanoparticles, and black carbon particles from air pollutants [39,40,41]. In this study, we detected MNPs larger than 300 nm in human urine. Although an in vivo mouse study has demonstrated that both 100 nm and 3 μm NMPs can be excreted in urine [24], and MNPs between 0.22 and 5 μm have been detected in human urine [16,20,27], a key unresolved question remains: Can these MNPs truly be excreted via urine? Given that the glomerular filtration barrier (GFB) has an estimated cutoff size of approximately 10 nm [42], the potential excretion of larger plastic particles remains controversial. In nature, certain large particles have been shown to undergo renal clearance. For instance, herpes simplex virus (HSV-1) and cytomegalovirus (CMV), both enveloped icosahedral double-stranded DNA viruses, have been detected in urine despite their relatively large size (150–240 nm) [43,43,44,45]. Based on available research evidence and the detection results of this study, we propose that plastic particles larger than 10 nm, including those in the micrometer range, may undergo urinary excretion. Firstly, secretion and organelle-extrusion by proximal tubular epithelial cells (PTECs) are highly likely to play a significant role in the urinary excretion of MNPs [46,47,48,49]. Secondly, damage to the glomerular basement membrane (GBM) increases the permeability of the GFB, potentially allowing large-sized particulate matter to pass into the urine [50]. However, more robust evidence is still required to validate this proposed mechanism. In future studies, under the premise of rigorous analytical quality control, urinary β2-microglobulin—a well-established biomarker of GBM damage—could serve as a key indicator to verify whether the presence of MNPs in urine is associated with compromised glomerular filtration function. In addition, carboxylated PS microspheres with a particle size of 25 nm are distributed in a cluster-like morphology of 10–25 μm in multiple tissues after entry into SD rats [51], so it is also highly likely that MNPs detected in the urine are formed by the repolymerization of small-sized MNPs in the urine after filtration with the glomerulus.

The detection of MNPs in human urine using mass spectrometry remains a developing research area. Current methods for analyzing MNPs in urine have limitations in terms of sensitivity, resolution, and quantitative ability, especially when dealing with complex urine matrices. Spectroscopic techniques are suitable for qualitative analysis but have limited effectiveness for small-sized particles; thermal analysis techniques combined with GC/MS have high quantitative potential but need to be improved to minimize the impact of matrix effects and signal fluctuations in the mass spectrometer. The strengths and weaknesses of these methods suggest that the development of a sensitive, stable, and standardized analytical method is important for the study of MNPs in human urine. A limited number of studies have employed Py-GC/MS or TD-GC/MS for the detection of MNPs in human urine, employing various sample pretreatment methods [20,22]. However, external standard quantification may lead to potential inaccuracies arising from matrix effects and signal variability. A major strength of this study is the establishment of a quantitative Py-GC/MS method incorporating a ^13^C-labeled polymer as an internal standard for the analysis of MNPs in complex biological matrices, specifically human urine. The use of isotopically labeled standards enables effective correction for matrix-induced signal variations, thereby enhancing both the accuracy and reproducibility of the quantification results. Furthermore, the method was subjected to rigorous validation to confirm its analytical robustness and reliability under biologically relevant conditions. The recovery rates of different polymers in this study showed notable variability, with lower recovery for PP and slight over-recovery for PET at low concentrations. This variability reflects a broader challenge in microplastic analysis: The morphology, size, and surface properties of MNPs—critical factors affecting recovery—are largely unknown in real biological samples. Unlike dissolved chemicals, MNPs are present in diverse and often unpredictable forms, making it difficult to simulate their behavior through standard spiking experiments. In our study, the test particles were prepared by drying polymer solutions in dichloromethane, and their resulting morphology could have influenced the filtration efficiency. Therefore, current recovery experiments should be interpreted as methodological references rather than precise indicators of true recovery. Future research should explore more representative control models and advanced imaging techniques to better characterize MNP behavior during sample preparation.

The number of MNP types detected varies across studies, with previous studies reporting 3–7 distinct MNPs in urine samples, while our study identified four MNP types. Our results demonstrated that LDPE and PET had the highest detection rate in human urine, followed by PS and PP, a trend consistent with Leslie et al.’s study on the detection of MNPs in human blood [18]. Additionally, we compared the detected urinary concentration levels of MNPs across studies. The average mass concentration of MNPs detected in urine in the current study was nearly 30-fold lower than previously reported concentrations measured by mass spectrometry-based methods in the literature, as shown in Table 5. The comparatively lower MNP concentrations observed in this study may be attributed to several factors. First, we used Py-GC/MS, which differs from TD-GC/MS employed in some other studies and may result in different detection efficiencies. Second, our pretreatment method involved physical filtration using membranes with a 300 nm pore size, whereas other studies adopted organic solvent extraction methods or membranes with smaller pore sizes that might recover a broader size range of particles, including those <300 nm. Additionally, differences in participant exposure profiles—such as indoor vs. outdoor environments, dietary habits, and geographical locations—could also contribute to the variation in MNP levels. Moreover, the significantly lower concentrations observed here may also result from the rigorous use of internal standard calibration in our analytical workflow, which effectively reduces matrix effects and mitigates potential overestimations of MNP concentrations. Thus, our findings highlight the critical importance of employing internal standards and appropriate calibration strategies to accurately quantify MNPs, thereby providing more realistic assessments of human exposure through urinary biomonitoring.

Ingestion via the digestive and respiratory tracts is recognized as the primary route of human exposure to MNPs [52,53]. To investigate the specific impact of dietary and behavioral factors, we analyzed the correlation between injection use, exercise frequency, food type, water intake, drinking water type, and the detection of MNPs in urine. The results demonstrated that the detection levels of LDPE, which collectively accounted for 67.72% of the total MNPs, were significantly positively correlated with participants’ daily drinking water type. Although PP accounts for a small proportion of total MNPs (7.06%), its detected amounts exhibit a close correlation with the frequency of teabag usage. After stratification, only the type of drinking water remained significantly associated with urinary LDPE levels (*p* = 0.035). These results suggested bottled water and teabags as a major source of MNPs in urine. MNP contamination in bottled drinking water may come from the source water, the production process, or the plastic packaging. Currently, PET is the primary material used for bottled drinking water packaging, while other plastics, such as PE and PC, may also be utilized for specific applications [54]. A study analyzed 69 plastic bottled drinking water samples from 23 brands available in the Chinese market using focal plane array-based micro-Fourier transform infrared microscopy imaging and identified 11 types of polymers. Among these, aside from cellulose, the most prevalent plastic polymers were PET (7%), PE (6%), PS (5%), and PA (4%) [55]. Qian et al. developed a data science-driven hyperspectral stimulated Raman scattering (SRS) microscopy technique, enabling the statistical analysis of individual plastic particles as small as 100 to 200 nm. Using this technique, an analysis of bottled water estimated the MNP concentration at approximately (2.4 ± 1.3) × 10^5^ particles/L, with NPs smaller than 1 μm comprising approximately 90% of the total particles [56]. A simulated study showed that various teabag materials release MNPs into the aqueous phase during typical use, and PP exhibits higher release amounts than nylon [57]. The release mechanism is attributed to thermal stress from hot water (e.g., 95 °C), which causes the structural degradation of plastic polymers, leading to the detachment of particles with diameters ranging from nanometers to micrometers [58]. These findings indicate that bottled drinking water and teabags seem to be significant sources of human exposure to small-sized NPs.

Although small-sized NPs are often overlooked in conventional spectroscopic detection and mass quantification due to low detection sensitivity or minimal mass contribution, their potential toxicity and health effects should not be underestimated. NPs can cross biological barriers more readily than MPs due to their smaller size and unique physicochemical properties, potentially leading to greater toxicity and health risks. Studies have demonstrated that NPs can penetrate multiple biological barriers, including the gastrointestinal barrier, placental barrier and the blood–brain barrier, allowing their accumulation in vital organs such as the brain, liver, lungs, and circulatory system [2,59,60]. Furthermore, the high specific surface area of NPs enables them to adsorb and transport more environmental contaminants, including metals and pharmaceuticals, thereby amplifying their toxic effects [61].

This study has several limitations that should be acknowledged. First, due to technical constraints associated with physical filtration separation, our method was only able to detect MNPs with particle sizes ≥ 300 nm. As a result, smaller nanoparticles (< 300 nm), which may have a greater capacity to cross biological barriers and pose higher health risks, could not be captured. Future studies should prioritize the development of more advanced separation and detection techniques to include this critical size fraction. Second, while our method achieved relatively low detection limits compared to prior studies, further improvements in analytical sensitivity are needed—particularly for the accurate quantification of low-abundance MNPs. Enhancing sensitivity will be essential for advancing research on low-level exposure and understanding the full extent of human health risks associated with MNPs. This study included only 18 participants, most of whom were female and within a narrow age range (20–28 years), with similar occupational and environmental backgrounds, as all were students or staff working in indoor academic settings. While this relatively controlled population helps to minimize confounding variables, it also limits the generalizability of our findings. The exposure patterns and microplastic profiles observed in this group may not fully represent those found in the general population with more diverse demographics, lifestyles, and environmental exposures. Therefore, further studies involving larger and more heterogeneous populations are necessary to validate and extend the conclusions drawn from this study.

There are at least two major challenges in using urine to assess human exposure to MNPs in future studies. First, the optimal urine sampling time point for assessing internal MNP exposure remains uncertain due to the limited understanding of the MNP metabolism and elimination kinetics in the human body. Urine biomonitoring can provide insights into individual and population-level exposure, but its interpretation must consider half-life data. Whether MNP detection in single-spot urine reflects recent exposure or long-term accumulation remains unclear and necessitates further studies on the elimination properties (ADME) of MNPs in humans. Second, the insoluble nature of MNPs and their non-homogeneous distribution in biological fluids pose a significant challenge to exposure assessment accuracy. This issue arises from the physical properties of MNPs and sample variability, rather than analytical techniques. Studies on MNPs in human blood have shown high variability in duplicate blood samples collected simultaneously from the same individual [18]. Similarly, MNPs in urine may exhibit heterogeneous distribution, leading to inconsistent results. To improve exposure assessment reliability, we recommend that future studies collect multiple urine samples from the same individual at different time points and analyze them separately or as pooled samples. This approach could provide a more comprehensive evaluation of individual MNP exposure, minimizing variability and enhancing data accuracy.

## 5. Conclusions

This study provided quantitative evidence of human internal exposure toMNPs by developing and validating an effective analytical method based on a stable isotope internal standard utilizing Py-GC/MS for detection in non-invasive urine samples. MNPs (≥300 nm) were universally detected in all analyzed samples, with LDPE being the predominant polymer type, indicating widespread internal exposure and suggesting potential contributions originating from drinking water sources. While these findings underscore the ubiquity of MNPs in human biofluids, the mechanisms underlying their urinary excretion remain poorly understood, thus requiring further rigorous investigation. Overall, the analytical method developed in this study provides a robust tool for assessing human exposure to plastic, thereby emphasizing the critical need for future research aimed at elucidating their potential health implications.

## Figures and Tables

**Table 1:** Spiking Recovery for Four Target Polymers Identified and Quantified by Py-GC/MS.

Polymers	Low-Dose Spiked (n = 3)		High-Dose Spiked (n = 3)	
	Recovery, %	RSD, %	Recovery, %	RSD, %
PET	117.64 ± 6.64	5.64	85.95 ± 13.43	15.62
PP	59.84 ± 3.09	5.16	56.73 ± 17.64	31.09
LDPE	96.16 ± 11.38	11.83	78.81± 0.68	0.86
PS	63.24 ± 9.91	15.67	69.51 ± 7.89	11.35

**Table 2:** Detection of MNPs (μg) in procedural blank controls.

Detection (μg)	PET	PP	LDPE	PS
Blank 1	n.d.	0.015	0.051	0.024
Blank 2	n.d.	n.d.	0.065	0.047
Blank 3	n.d.	n.d.	0.039	0.023
Blank 4	n.d.	0.019	n.d.	0.047
Blank 5	n.d.	n.d.	0.037	0.055
Blank 6	n.d.	0.025	0.079	0.131
Blank 7	n.d.	0.016	0.016	0.086
Blank 8	n.d.	0.015	n.d.	0.039
Mean	N/A	0.011	0.036	0.057
SD	N/A	0.010	0.029	0.036

n.d.: not detectable; N/A: not available.

**Table 3:** IDLs, IQLs, LODs, and LOQs (ng) for Four Target Polymers.

Polymers	PET	PP	LDPE	PS
IDL	0.66	0.10	6.94	0.52
IQL	2.19	0.32	23.13	1.73
LOD	0.66	29.23	87.36	108.49
LOQ	2.19	96.47	288.29	358.03

**Table 4:** Stratified analysis (with gender and BMI as control variables).

Polymers	Bottled Water Consumption Frequency		Drinking Water Type	
	*r*	*p*	*r*	*p*
LDPE	0.382	0.144	0.533	0.033
**Polymers**	**Teabag usage frequency**			
	***r***	***p***		
PP	0.307	0.248		

**Table 5:** Comparison of MNP concentrations in human urine across studies.

Study	Sample Pretreatment Method	Detection Method	Types of Polymers Detected	Detection Frequency %	Concentration
Zhu et al. [20]	Membrane filtration	TD-GC/MS	PVC PE PS PP PET **Total**	**100.0**	**6.49 ± 4.56 ^a^**
Song et al. [21]	Extraction with various organic solvents (chloroform, hexafluoroisopropanol, xylene)	Py-GC/MS	PS PE PP PMMA PVC PET PA66 **Total**	92.3 88.5 57.7 3.8 96.2 42.3 26.9 **100.0**	0.10 (0.09–0.15) ^b^ 1.18 (1.01–1.52) 0.09 (0.00–0.14) 0.00 (0.00–0.00) 0.80 (0.65–1.34) 0.00 (0.00–4.80) 0.00 (0.00–1.67) **5.06 (2.21–8.61)**
Song et al. [22]	Extraction with various organic solvents (chloroform, hexafluoroisopropanol, xylene)	Py-GC/MS	PE PVC PA66 **Total**	75.0 58.3 16.7 **75.0**	1.44 (0.38–2.52) ^b^ 0.03 (0.00–0.15) 0.00 (0.00–0.00) **1.51 (0.40–2.71)**
This Study	Membrane filtration	Py-GC/MS	LDPE PS PET PP **Total**	100.0 55.6 100.0 44.4 **100.0**	**0.268 ± 0.235 ^a^**

^a^ mean ± SD, μg/mL. ^b^ median (interquartile range), μg/g.

## Data Availability

The data that support the findings of this study are available from the corresponding author upon reasonable request.

## Supplementary Materials

The following supporting information can be downloaded at https://www.mdpi.com/article/10.3390/toxics13060452/s1, Table S1: Parameters of accelerated solvent extraction (ASE). Text S1: Inclusion and exclusion criteria; Table S2: Questionnaire; Table S3: Questionnaire information for all participants; Figure S1: Customized quartz filter connections (8 mm i.d.); Figure S2: Pyrolysis cup base; Table S4: Parameters for double-shot Py-GC/MS measurements; Table S5: Selection ion monitoring (SIM) properties; Table S6: Calibrations (*y* = relative peak area with regard to P(E-^13^C_2_), *x* = polymer concentration in μg); Figure S3: Mass spectra of thermal polymerization products four standards; Figure S4: Standard curve calibrated by internal standard method; Figure S5: Extracted ion chromatogram (EIC) of 4 MNPs and internal standard for samples.

## References

[1] Wilcox C., Van Sebille E., Hardesty B.D. (2016). Threat of plastic pollution to seabirds is global, pervasive, and increasing. Proc. Natl. Acad. Sci. USA.

[2] Wright S.L., Kelly F.J. (2017). Plastic and Human Health: A Micro Issue?. Environ. Sci. Technol..

[3] Kushwaha M., Shankar S., Goel D., Singh S., Rahul J., Rachna K., Singh J. (2024). Microplastics pollution in the marine environment: A review of sources, impacts and mitigation. Mar. Pollut. Bull..

[4] Yang L., Zhang Y.L., Kang S.C., Wang Z.Q., Wu C.X. (2021). Microplastics in soil: A review on methods, occurrence, sources, and potential risk. Sci. Total Environ..

[5] Zhang Q., Zhao Y.P., Du F.N., Cai H.W., Wang G.H., Shi H.H. (2020). Microplastic Fallout in Different Indoor Environments. Environ. Sci. Technol..

[6] Alfaro-Núñez A., Astorga D., Caceres-Farías L., Bastidas L., Villegas C.S., Macay K.C., Christensen J.H. (2022). Microplastic pollution in seawater and marine organisms across the Tropical Eastern Pacific and Galapagos. Sci. Rep..

[7] Zhang S., Wu H., Hou J. (2023). Progress on the Effects of Microplastics on Aquatic Crustaceans: A Review. Int. J. Mol. Sci..

[8] da Silva Brito W.A., Mutter F., Wende K., Cecchini A.L., Schmidt A., Bekeschus S. (2022). Consequences of nano and microplastic exposure in rodent models: The known and unknown. Part. Fibre Toxicol..

[9] van Raamsdonk L.W.D., van der Zande M., Koelmans A.A., Hoogenboom R., Peters R.J.B., Groot M.J., Peijnenburg A., Weesepoel Y.J.A. (2020). Current Insights into Monitoring, Bioaccumulation, and Potential Health Effects of Microplastics Present in the Food Chain. Foods.

[10] Wu P., Lin S., Cao G., Wu J., Jin H., Wang C., Wong M.H., Yang Z., Cai Z. (2022). Absorption, distribution, metabolism, excretion and toxicity of microplastics in the human body and health implications. J. Hazard. Mater..

[11] Zhang X.Y., He Y.C., Xie Z.Y., Peng S.H., Xie C.G., Wang H.T., Liu L., Kang J., Yuan H.P., Liu Y. (2022). Effect of microplastics on nasal and gut microbiota of high-exposure population: Protocol for an observational cross-sectional study. Medicine.

[12] Krafft C., Popp J., Bronsert P., Miernik A. (2023). Raman Spectroscopic Imaging of Human Bladder Resectates towards Intraoperative Cancer Assessment. Cancers.

[13] Guo X.L., Wang L., Wang X.Y., Li D.B., Wang H., Xu H.F., Liu Y., Kang R.H., Chen Q., Zheng L.Y. (2024). Discovery and analysis of microplastics in human bone marrow. J. Hazard. Mater..

[14] Guan Q.Q., Jiang J., Huang Y., Wang Q., Liu Z.F., Ma X., Yang X.A., Li Y., Wang S.Q., Cui W.D. (2023). The landscape of micron-scale particles including microplastics in human enclosed body fluids. J. Hazard. Mater..

[15] Jenner L.C., Rotchell J.M., Bennett R.T., Cowen M., Tentzeris V., Sadofsky L.R. (2022). Detection of microplastics in human lung tissue using muFTIR spectroscopy. Sci. Total Environ..

[16] Zhang C., Zhang G., Sun K., Ren J., Zhou J., Liu X., Lin F., Yang H., Cao J., Nie L. (2024). Association of mixed exposure to microplastics with sperm dysfunction: A multi-site study in China. eBioMedicine.

[17] Liu S., Liu X., Guo J., Yang R., Wang H., Sun Y., Chen B., Dong R. (2023). The Association Between Microplastics and Microbiota in Placentas and Meconium: The First Evidence in Humans. Environ. Sci. Technol..

[18] Leslie H.A., van Velzen M.J.M., Brandsma S.H., Vethaak A.D., Garcia-Vallejo J.J., Lamoree M.H. (2022). Discovery and quantification of plastic particle pollution in human blood. Environ. Int..

[19] Malafaia G., Barceló D. (2023). Microplastics in human samples: Recent advances, hot-spots, and analytical challenges. TrAC Trends Anal. Chem..

[20] Zhu L., Wu Z.X., Dong J., Zhao S.Y., Zhu J.Y., Wang W.P., Ma F.J., An L.H. (2024). Unveiling Small-Sized Plastic Particles Hidden behind Large-Sized Ones in Human Excretion and Their Potential Sources. Environ. Sci. Technol..

[21] Song Y., Zhang J., Yang L., Huang Y., Zhang N., Ma G. (2024). Internal and external microplastic exposure in young adults: A pilot study involving 26 college students in Changsha, China. Environ. Res..

[22] Song X., Chen T., Chen Z., Du L., Qiu X., Zhang Y., Li Y., Zhu Y., Tan Z., Mo Y. (2024). Micro(nano)plastics in human urine: A surprising contrast between Chongqing’s urban and rural regions. Sci. Total Environ..

[23] Smith M., Love D.C., Rochman C.M., Neff R.A. (2018). Microplastics in Seafood and the Implications for Human Health. Curr. Environ. Health Rep..

[24] Sun W., Jin C.H., Bai Y.L., Ma R.X., Deng Y., Gao Y., Pan G.W., Yang Z.S., Yan L.J. (2022). Blood uptake and urine excretion of nano- and micro-plastics after a single exposure. Sci. Total Environ..

[25] Exacoustos O., Artini C., Massardo S., Caboni C., Pastorino A., Chiarenza S., Zaza G., Stallone G., Ghiggeri G.M., Angeletti A. (2023). First Identification and Characterization of Microplastics in Human Kidney and Urine. Nephrol. Dial. Transpl..

[26] Pironti C., Notarstefano V., Ricciardi M., Motta O., Giorgini E., Montano L. (2023). First Evidence of Microplastics in Human Urine, a Preliminary Study of Intake in the Human Body. Toxics.

[27] Rotchell J.M., Austin C., Chapman E., Atherall C.A., Liddle C.R., Dunstan T.S., Blackburn B., Mead A., Filart K., Beeby E. (2024). Microplastics in human urine: Characterisation using μFTIR and sampling challenges using healthy donors and endometriosis participants. Ecotoxicol. Environ. Saf..

[28] Schwabl P., Köppel S., Königshofer P., Bucsics T., Trauner M., Reiberger T., Liebmann B. (2019). Detection of Various Microplastics in Human Stool: A Prospective Case Series. Ann. Intern. Med..

[29] Jahedi F., Haghighi Fard N.J., Ahmadi M., Takdastan A., Shoushtari M.H., Dehbandi R., Turner A. (2025). Microplastics in urine, sputum and lung lavage fluid from patients with respiratory illnesses. Environ. Res..

[30] Kutralam-Muniasamy G., Shruti V.C., Pérez-Guevara F., Roy P.D. (2023). Microplastic diagnostics in humans: “The 3Ps” Progress, problems, and prospects. Sci. Total Environ..

[31] Song Y.K., Hong S.H., Eo S., Shim W.J. (2021). A comparison of spectroscopic analysis methods for microplastics: Manual, semi-automated, and automated Fourier transform infrared and Raman techniques. Mar. Pollut. Bull..

[32] Seeley M.E., Lynch J.M. (2023). Previous successes and untapped potential of pyrolysis-GC/MS for the analysis of plastic pollution. Anal. Bioanal. Chem..

[33] Ainali N.M., Kalaronis D., Kontogiannis A., Evgenidou E., Kyzas G.Z., Yang X., Bikiaris D.N., Lambropoulou D.A. (2021). Microplastics in the environment: Sampling, pretreatment, analysis and occurrence based on current and newly-exploited chromatographic approaches. Sci. Total Environ..

[34] Hermabessiere L., Rochman C.M. (2021). Microwave-Assisted Extraction for Quantification of Microplastics Using Pyrolysis-Gas Chromatography/Mass Spectrometry. Environ. Toxicol. Chem..

[35] Funck M., Yildirim A., Nickel C., Schram J., Schmidt T.C., Tuerk J. (2020). Identification of microplastics in wastewater after cascade filtration using Pyrolysis-GC–MS. MethodsX.

[36] Lauschke T., Dierkes G., Schweyen P., Ternes T.A. (2021). Evaluation of poly(styrene-d5) and poly(4-fluorostyrene) as internal standards for microplastics quantification by thermoanalytical methods. J. Anal. Appl. Pyrolysis.

[37] Rodland E.S., Samanipour S., Rauert C., Okoffo E.D., Reid M.J., Heier L.S., Lind O.C., Thomas K.V., Meland S. (2022). A novel method for the quantification of tire and polymer-modified bitumen particles in environmental samples by pyrolysis gas chromatography mass spectroscopy. J. Hazard. Mater..

[38] (2008). Capability of Detection Part 5: Methodology in the Linear and Non-Linear Calibration Cases.

[39] Ambiga N., Nagarajan A. (2023). Possibilities of using nano particles in human urine for transient biometrics. Mater. Today Proc..

[40] Saenen N.D., Bove H., Steuwe C., Roeffaers M.B.J., Provost E.B., Lefebvre W., Vanpoucke C., Ameloot M., Nawrot T.S. (2017). Children’s Urinary Environmental Carbon Load. A Novel Marker Reflecting Residential Ambient Air Pollution Exposure?. Am. J. Respir. Crit. Care Med..

[41] Parra-Sánchez M., Marcuello López A., García-Rey S., Zakariya-Yousef Breval I., Bernal Martínez S., Pueyo Rodríguez I., Martín-Mazuelos E., Palomares Folía J.C. (2016). Performance of the HSV OligoGen kit for the diagnosis of herpes simplex virus type 1 and 2. Diagn. Microbiol. Infect. Dis..

[42] Zuckerman J.E., Choi C.H.J., Han H., Davis M.E. (2012). Polycation-siRNA nanoparticles can disassemble at the kidney glomerular basement membrane. Proc. Natl. Acad. Sci. USA.

[43] Schottstedt V., Blümel J., Burger R., Drosten C., Gröner A., Gürtler L., Heiden M., Hildebrandt M., Jansen B., Montag-Lessing T. (2010). Human Cytomegalovirus (HCMV)—Revised. Transfus. Med. Hemother..

[44] Laine R.F., Albecka A., van de Linde S., Rees E.J., Crump C.M., Kaminski C.F. (2015). Structural analysis of herpes simplex virus by optical super-resolution imaging. Nat. Commun..

[45] Henry C., Hartsock R.J., Kirk Z., Behrer R. (1978). Detection of Viruria in Cytomegalovirus-infected Infants by Electron Microscopy. Am. J. Clin. Pathol..

[46] Wyss P.P., Lamichhane S.P., Abed A., Vonwil D., Kretz O., Huber T.B., Sarem M., Shastri V.P. (2020). Renal clearance of polymeric nanoparticles by mimicry of glycan surface of viruses. Biomaterials.

[47] Williams R.M., Shah J., Tian H.S., Chen X., Geissmann F., Jaimes E.A., Heller D.A. (2018). Selective Nanoparticle Targeting of the Renal Tubules. Hypertension.

[48] Naumenko V., Nikitin A., Kapitanova K., Melnikov P., Vodopyanov S., Garanina A., Valikhov M., Ilyasov A., Vishnevskiy D., Markov A. (2019). Intravital microscopy reveals a novel mechanism of nanoparticles excretion in kidney. J. Control. Release.

[49] Huang Y., Yu M., Zheng J. (2023). Proximal tubules eliminate endocytosed gold nanoparticles through an organelle-extrusion-mediated self-renewal mechanism. Nat. Nanotechnol..

[50] Lawrence M.G., Altenburg M.K., Sanford R., Willett J.D., Bleasdale B., Ballou B., Wilder J., Li F., Miner J.H., Berg U.B. (2017). Permeation of macromolecules into the renal glomerular basement membrane and capture by the tubules. Proc. Natl. Acad. Sci. USA.

[51] Cary C.M., DeLoid G.M., Yang Z., Bitounis D., Polunas M., Goedken M.J., Buckley B., Cheatham B., Stapleton P.A., Demokritou P. (2023). Ingested Polystyrene Nanospheres Translocate to Placenta and Fetal Tissues in Pregnant Rats: Potential Health Implications. Nanomaterials.

[52] De-la-Torre G.E. (2019). Microplastics: An emerging threat to food security and human health. J. Food Sci. Technol..

[53] Barboza L.G.A., Vethaak A.D., Lavorante B.R.B.O., Lundebye A.K., Guilhermino L. (2018). Marine microplastic debris: An emerging issue for food security, food safety and human health. Mar. Pollut. Bull..

[54] Mukhopadhyay M., Jalal M., Vignesh G., Ziauddin M., Sampath S., Bharat G.K., Nizzetto L., Chakraborty P. (2022). Migration of Plasticizers from Polyethylene Terephthalate and Low-Density Polyethylene Casing into Bottled Water: A Case Study From India. Bull. Environ. Contam. Toxicol..

[55] Xuejun Z., Jin W., Hongyan L., Huimin Z., Jiang H., Lei Z.D. (2021). Microplastic pollution of bottled water in China. J. Water Process Eng..

[56] Qian N., Gao X., Lang X., Deng H., Bratu T.M., Chen Q., Stapleton P., Yan B., Min W. (2024). Rapid single-particle chemical imaging of nanoplastics by SRS microscopy. Proc. Natl. Acad. Sci. USA.

[57] Banaei G., Abass D., Tavakolpournegari A., Martín-Pérez J., Gutiérrez J., Peng G., Reemtsma T., Marcos R., Hernández A., García-Rodríguez A. (2024). Teabag-derived micro/nanoplastics (true-to-life MNPLs) as a surrogate for real-life exposure scenarios. Chemosphere.

[58] Hernandez L.M., Xu E.G., Larsson H.C.E., Tahara R., Maisuria V.B., Tufenkji N. (2019). Plastic Teabags Release Billions of Microparticles and Nanoparticles into Tea. Environ. Sci. Technol..

[59] Mitrano D.M., Wick P., Nowack B. (2021). Placing nanoplastics in the context of global plastic pollution. Nat. Nanotechnol..

[60] Li P., Liu J. (2024). Micro(nano)plastics in the Human Body: Sources, Occurrences, Fates, and Health Risks. Environ. Sci. Technol..

[61] Gigault J., El Hadri H., Nguyen B., Grassl B., Rowenczyk L., Tufenkji N., Feng S., Wiesner M. (2021). Nanoplastics are neither microplastics nor engineered nanoparticles. Nat. Nanotechnol..

