# Supplementary material for: Quantitative Detection of Micro- and Nanoplastics (≥300 nm) in Human Urine Using Double-Shot Py-GC/MS with Internal Standard Calibration
Source: Toxics. 2025 May 29;13(6):452. doi: 10.3390/toxics13060452 (PMC12197141; doi:10.3390/toxics13060452)
Supplement: Supplementary file 1 [file toxics-13-00452-s001.zip › toxics-3606279-supplementary.pdf]

Supporting Information

Quantitative Detection of Micro- and Nanoplastics (⩾ 300 nm) in Human Urine Using Double-shot Py-GC/MS with Internal Standard Calibration

Shanshan Ji [1], Wei Wang [2], Yong Wang [2], Hexiang Bai [1], Zhuo Li [3], Zongli Huo [4], and Kai Luo [1]**

[1] Key Laboratory of Environmental Medical Engineering, Ministry of Education, School of Public Health, Southeast University, Nanjing, 210009, China

[2] Institute of Forensic Science and Technology of Nanjing Public Security Bureau, Nanjing 210001, China

[3] Department of Emergency, Children's Hospital of Nanjing Medical University, Nanjing, 210008, China

[4] Jiangsu Provincial Center for Disease Control and Prevention, Nanjing, 210009, China

Summary: The supplementary information includes one text, seventables and five figures in twelve pages.

Contents

Table S1. Parameters of accelerated solvent extraction (ASE).
Text S1. Inclusion and exclusion criteria.
Table S2. Questionnaire.
Table S3. Questionnaire information for all participants.
Figure S1. Customized quartz filter connections (8mm i.d.).
Figure S2. Pyrolysis cup base.
Table S4. Parameters for double shot Py-GC/MS measurements.
Table S5. Selection ion monitoring (SIM) properties.
Table S6. Calibrations (y = relative peak area with regard to P(E-$^{13}C_2$), x = polymer concentration in μg)
Figure S3. Mass spectra of thermal polymerization products four standards.
Figure S4. Standard curve calibrated by internal standard method.
Figure S5. Extracted ion chromatogram (EIC) of 4 MNPs and internal standard for samples.

Table S1. Parameters of accelerated solvent extraction (ASE).

Parameter	Pre-extraction (clean up)	Microplastic extraction	Post-extraction
Extraction solvent	Methanol	Dichloromethane	Dichloromethane
Extraction temperature (℃)	100	180	180
Heating time (min)	5	9	9
Extraction pressure (psi)	1500	1500	1500
Static time	5 s	5 min	5 min
Cycles	3	3	1
Rinse volume (%)	45	80	80
Purge time (min)	1.25	1.25	1.25
System rinse volume (mL)	29.7	52.8	17.6

Text S1. Inclusion and exclusion criteria.

The inclusion criteria were as follows: (1) subjects who voluntarily participated in the study and signed an informed consent form; (2) male or female aged 18-65 years; (3) no serious primary diseases of the cardiovascular, digestive, urinary, or hematopoietic systems; (4) participants resided in the area for more than 20 hours per day continuously for one year. The exclusion criteria were as follows: (1) respiratory system infection within three months; (2) smoking history; (3) pregnancy or breastfeeding; (4) involvement in high-risk industries related to plastic pollution.

Table S2. Questionnaire.

Questions	Options
Basic information	Gender
	Age
	Height
	Weight
Have you had any recent acute respiratory infections (sneezing, runny nose, cough, sore throat, fever)?	Yes
	No
Have you had any history of injections, infusions or surgical treatments in the last year?	Yes
	No
Do you suffer from any of the following conditions?	Hypertension
	Hyperglycaemia
	Hyperlipidaemia
	Digestive system (pharynx, oesophagus, stomach, intestines) diseases
	Urinary system (kidneys, ureters, bladder, urethra) diseases
	None
How often do you exercise?	Never
	Occasionally
	1-3 days
	4-7 days
	8-15 days
How long do you exercise each time?	< 1 hour
	1-2 hours
	2-4 hours
	> 4 hours
How often do you drink alcohol during the week?	Never
	1-2 times
	3-6 times
	7-14 times
	> 14 times
What type of alcohol do you drink?	Beer
	Other (red wine, white wine, yellow wine)
Do you usually cook for yourself?	Never or seldom
	Often
How often do you eat takeaway food in a week?	Never
	1-2 times
	3-6 times
	> 6 times
How often do you consume each of the following foods in a week?	

Plastic bottled water/drinks (e.g. cola etc.)	_______ times a week
Take-away milk tea/coffee/tea made from tea bags	_______ times a week
Fish, seafood	_______ times a week
How much water do you drink every day?	< 500 mL
	500-1000 mL
	1000-1500 mL
	> 1500mL
	Unscheduled
How do you usually drink water?	Boiled water
	Drinking water filtration fountain
	Bottled water
What is the material of the cups/tableware you use most frequently? (√ for cups/ ○ for tableware)	Plastic
	Ceramic
	Stainless steel
	Glass
	Diamine imitation ceramic (commonly used in restaurants)
	Others

Table S3. Questionnaire information for all participants.

NO. Sample	Age	Gender	BMI	Injections, infusions, & surgeries LY	Exercise frequency, h/week	Alcohol consumption	Takeout-food intake, time/week	Bottled beverage consumption, time/week	Teabag usage, time/week	Aquatic product consumption, time/week	Drinking water intake, mL/d	Drinking water type	Tableware/Cups Material
#1	22	female	21.97	yes	4-6	yes	1-2	3	1	1	1000-1500	bottled water	ceramics, glass
#2	20	female	34.84	no	0-2	yes	3-6	0	3	2	1000-1500	boiled water	ceramics
#3	21	female	30.48	no	0-2	yes	3-6	2	6	0	> 1500	bottled water	plastics, stainless steel, glass, melamine, paper
#4	20	female	18.69	no	0-2	no	1-2	2	0	0	500-1000	purified water	stainless steel, melamine
#5	21	female	19.00	no	2-4	no	3-6	3	1	1	—	bottled water	glass
#6	23	male	21.80	no	2-4	no	3-6	7	1	0	—	purified water	paper
#7	23	male	24.58	no	2-4	no	1-2	7	0	2	—	purified water	plastics, stainless steel, glass
#8	24	male	22.79	no	0-2	no	3-6	7	1	0	—	bottled water	plastics, paper
#9	24	female	23.42	no	0	no	3-6	0	2	2	> 1500	boiled water	stainless steel, glass, melamine
#10	23	female	19.05	no	0-2	no	1-2	0	3	0	> 1500	purified water	glass, melamine
#11	26	female	21.67	no	0-2	yes	3-6	3	0	0	—	purified water	glass
#12	28	female	22.59	no	0	no	3-6	1	5	1	500-1000	purified water	Plastics, glass
#13	25	female	21.48	no	0	no	>7	2	1	0	500-1000	purified water	stainless steel
#14	24	female	17.97	no	2-4	no	1-2	2	1	4	1000-1500	purified water	stainless steel
#15	24	female	19.23	no	0-2	no	3-6	2	2	1	1000-1500	purified water	ceramics, melamine
#16	26	female	19.20	no	2-4	no	3-6	0	3	2	1000-1500	boiled water	glass
#17	24	female	20.70	yes	0-2	no	3-6	3	2	2	1000-1500	purified water	glass
#18	26	male	27.78	no	4-6	yes	3-6	1	1	1	1000-1500	boiled water	ceramics, melamine

There are five missing values in the water intake. Through Little's MVAR test in SPSS, it is found that the missing values are of the type of Missing Completely at Random (MCAR, $p = 0.755$). The mode imputation method is adopted to fill in the missing values, which is set as 1000-1500mL.

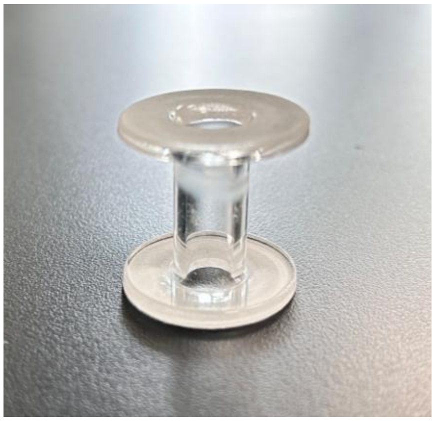

Figure S1. Customized quartz filter connections (8mm i.d.).

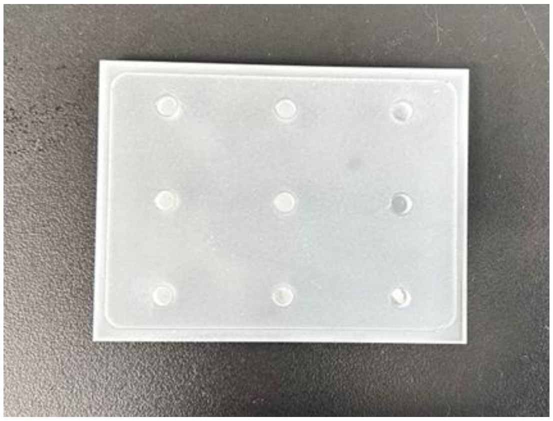

Figure S2. Pyrolysis cup base.

Table S4. Parameters for double shot Py-GC/MS measurements.

**Micro-furnace pyrolyzer**	
**Carrier gas**	**Helium**
**First shot**	
Pyrolysis temperature	100℃ → 300℃（50℃/min）
Interface temperature	200℃
Pyrolysis time	9.10 min
**Second shot**	
Pyrolysis temperature	600℃
Interface temperature	200℃
Pyrolysis time	5 min
**Gas chromatograph**	
Column	DB-5MS(J&W); 30 m, 0.25 mm i.d., 0.25 μm film thickness
Transfer line temperature	230℃
**First stage**	
Injector port temperature	300℃
Temperature program	40℃ → 230℃ (20℃/min) → 320℃（50℃/min）
Injection mode	Split mode (1:15 split ratio)
**Second stage**	
Injector port temperature	300℃
Temperature program	40℃ (2 min) →320℃（20℃/min, 2 min）
Injection mode	Split mode (1:15 split ratio)
**Mass spectrometer**	
Ionization energy	70 eV
Ion source temperature	230℃

Table S5. Selection ion monitoring (SIM) properties.

Polymer type	Characteristic decomposition products	Ions (Quantifier underlined)	Retention time (min)
PET (Tetramethylammonium hydroxide)	Dimethyl terephthalate	135, 163, 194	9.81 (First stage)<br>10.25 (Second stage)
PP	2,4-Dimethyl-1-heptene	70, 83, 126	2.70
LDPE	Cetene	69, 83,224	10.64
PS<br>Styrene polymers	Styrene	51, 78, 104	4.12
P(E-$^{13}C_2$)	–	89, 104, 119	5.85

Table S6. Calibrations (y = relative peak area with regard to P(E-$^{13}C_2$), x = polymer concentration in µg)

Polymer	Calibration functions	Linear range		Linearity (R^2)	Inter day (n=3) RSD (%)	Trial period (n=4) RSD (%)
		min (µg)	max (µg)			
PET	$y = 404.43x - 59.8950$	0.022	2.183	0.9771	6.50	12.28
PP	$y = 4.73x + 0.1654$	0.038	3.840	0.9970	6.64	7.13
LDPE	$y = 3.0455x + 0.1326$	0.031	3.100	0.9926	7.90	15.12
PS	$y = 68.564x - 7.3257$	0.032	3.200	0.9954	20.68	18.60

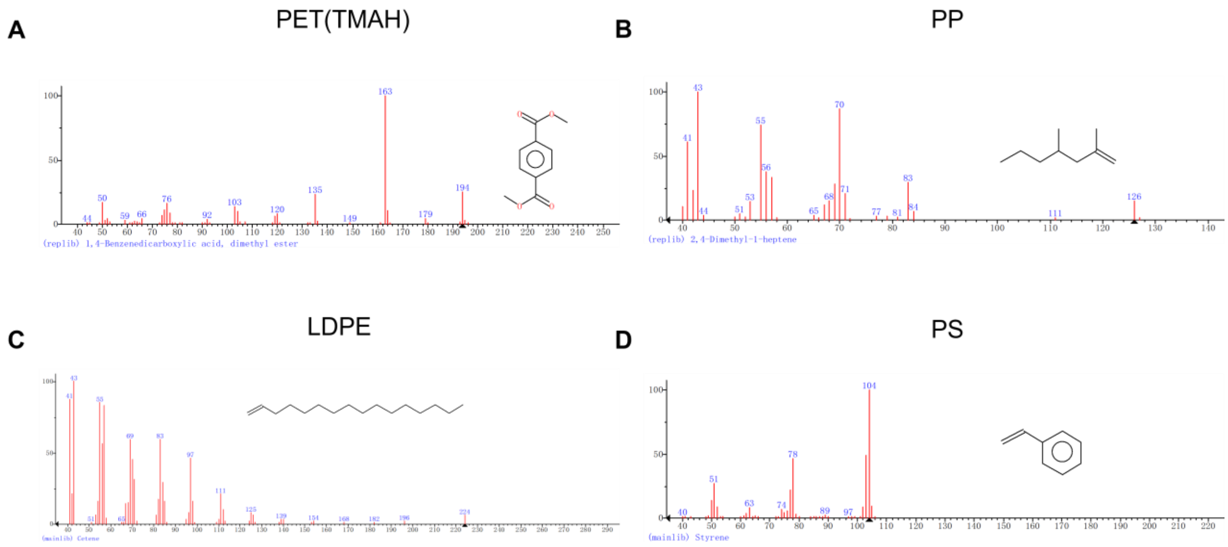

Figure S3. Mass spectra of thermal polymerization products four standards. (A) Dimethyl terephthalate – the thermal polymerization of PET under tetramethylammonium hydroxide derivatization (TMAH). (B) 2,4-Dimethyl-1-heptene – the thermal polymerization of PP. (C) Cetene– the thermal polymerization of LDPE. (D) Styrene – the thermal polymerization of PS.

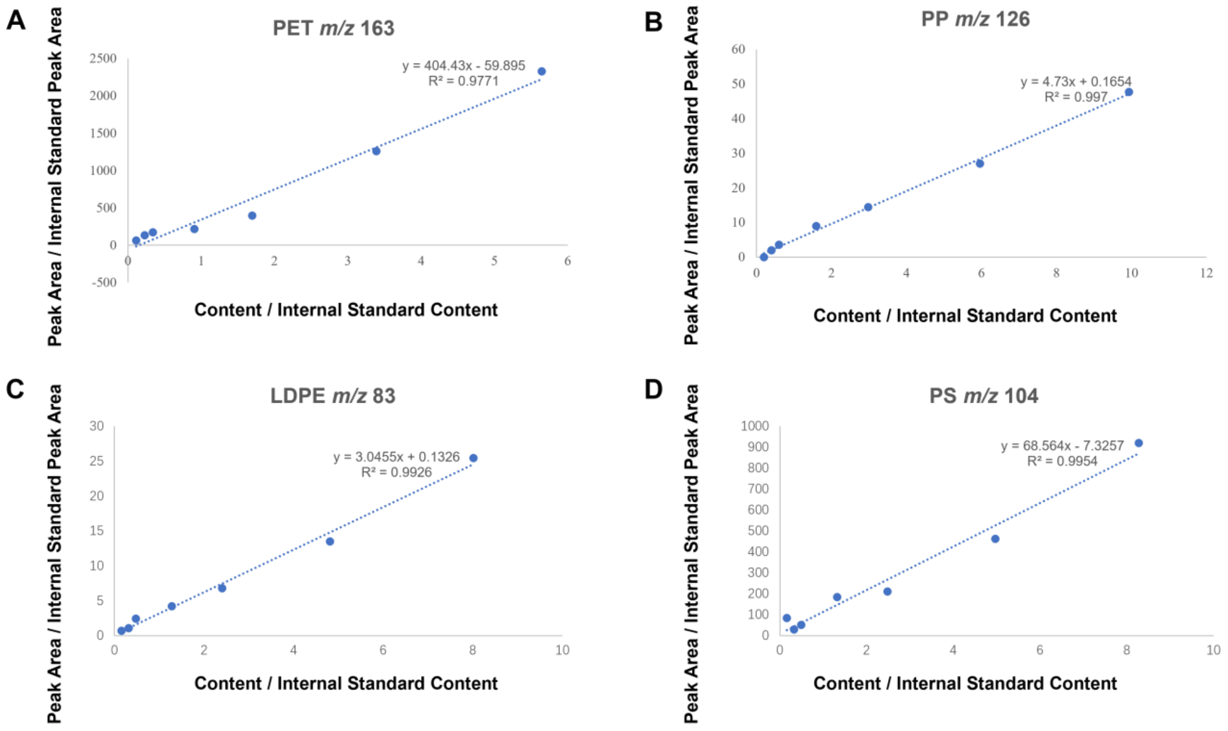

Figure S4. Curves calibrated by internal standard method.

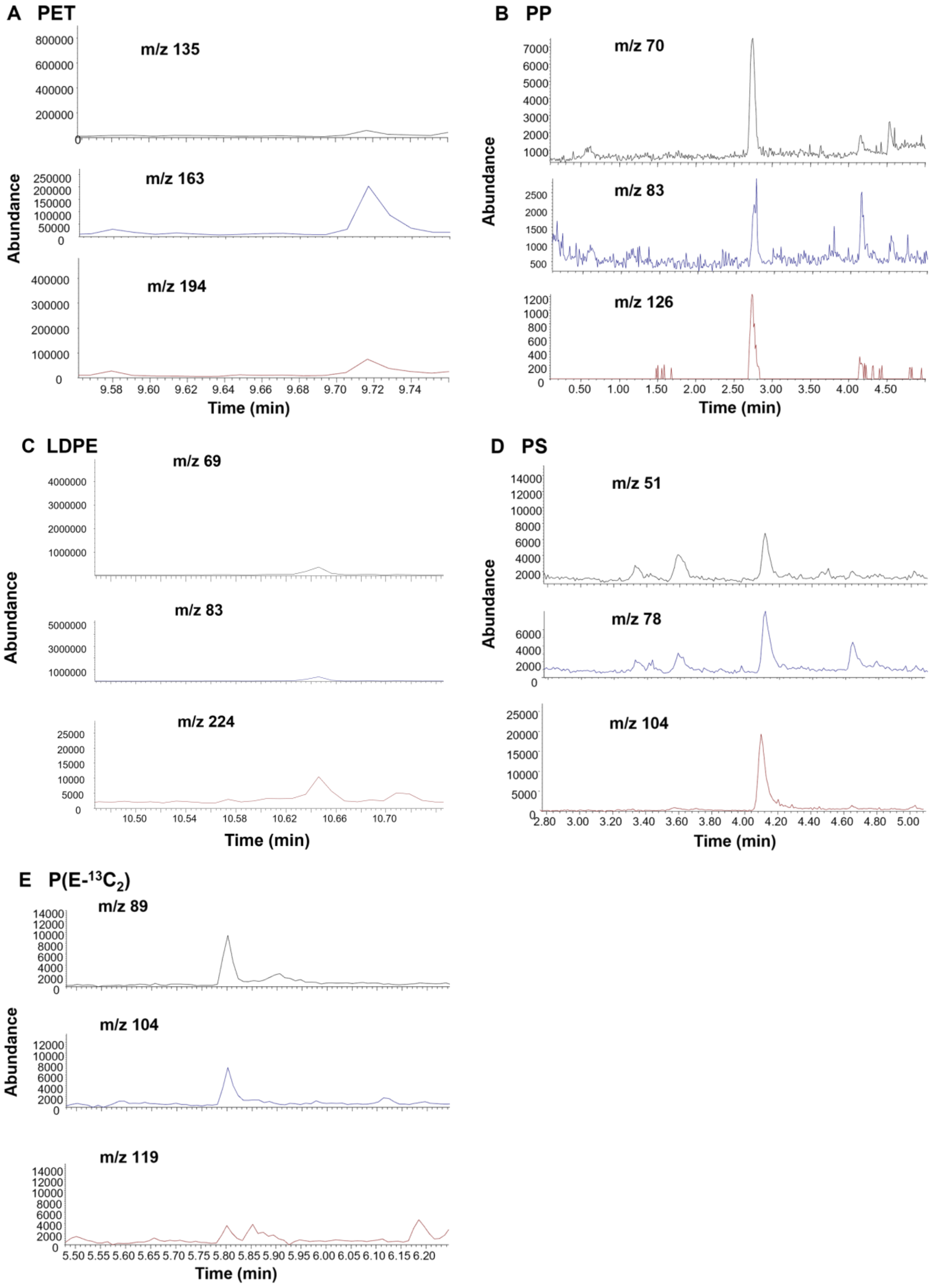

Figure S5. Extracted ion chromatogram (EIC) of 4 MNPs and internal standard for samples.
